# Quantitative analysis of 3D food printing layer extrusion accuracy: Contextualizing automated image analysis with human evaluations: Quantifying 3D food printing accuracy

**DOI:** 10.1016/j.crfs.2023.100511

**Published:** 2023-05-05

**Authors:** Yizhou Ma, Jelle Potappel, Maarten A.I. Schutyser, Remko M. Boom, Lu Zhang

**Affiliations:** Laboratory of Food Process Engineering, Wageningen University and Research, P.O. Box 17, 6700 AA, Wageningen, the Netherlands

**Keywords:** Direct ink writing, Consumer insights, Extrusion-based 3D food printing, Process monitoring

## Abstract

3D food printing can customize food appearance, textures, and flavors to tailor to specific consumer needs. Current 3D food printing depends on trial-and-error optimization and experienced printer operators, which limits the adoption of the technology by general consumers. Digital image analysis can be applied to monitor the 3D printing process, quantify printing errors, and guide optimization of the printing process. We here propose an automated printing accuracy assessment tool based on layer-wise image analysis. Printing inaccuracies are quantified based on over- and under-extrusion with reference to the digital design. The measured defects are compared to human evaluations via an online survey to contextualize the errors and identify the most useful measurements to improve printing efficiency. The survey participants marked oozing and over-extrusion as inaccurate printing which matched the results obtained from automated image analysis. Although under-extrusion was also quantified by the more sensitive digital tool, the survey participants did not perceive consistent under-extrusion as inaccurate printing. The contextualized digital assessment tool provides useful estimations of printing accuracy and corrective actions to avoid printing defects. The digital monitoring approach may accelerate the consumer adoption of 3D food printing by improving the perceived accuracy and efficiency of customized food printing.

## Highlights

• Layer-wise image analysis is automated to quantify 3D printing accuracy of foods.
• Digital and manual evaluations both indicate over-extrusion as inaccurate printing.
• Consistent under-extrusion is not perceived as inaccurate printing by human evaluators.
• Digital printing assessment can guide corrective actions of 3D food printing.

## Graphical abstract

**Figure:**
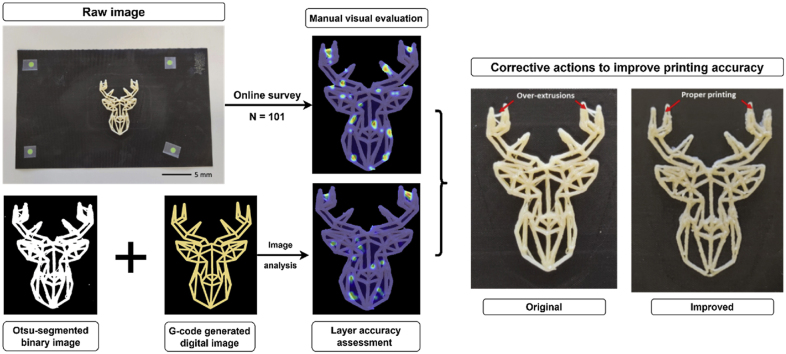

## 1 Introduction

3D food printing offers design flexibility to create personalized foods with customized appearances and sensory properties. Food materials such as chocolate, purees, and cookie dough have been printed into customized shapes that can appeal to specific consumer cohorts (Lanaro et al., 2017; Pulatsu et al., 2020). By building food structures layer-by-layer, 3D food printing can also modify food texture properties such as hardness and firmness by controlling the infill patterns and percentages of the designed food structures (Derossi et al., 2021; Fahmy et al., 2021). Suitable food textures have been designed and printed for consumers with special dietary needs (e.g. dysphagia patients and people with swallowing difficulties) (Pant et al., 2021). The flavor perception of 3D-printed foods can also be altered, for example by changing the position of the flavor-containing materials to create differences in sweetness and saltiness perception in chocolate waffles and starch-based snacks (Fahmy et al., 2021; Zhu et al., 2020). 3D food printing therefore introduces new opportunities to create customized foods for individual consumer needs.

Although 3D food printing is already possible, printed foods often suffer from poor printing accuracy (i.e. the printed object does not accurately resemble the digital design). Common printing inaccuracies include over- and under-extrusion of printing layers, layer shrinking and/or relaxation, and structural instability. Previous studies have focused on assessing food material printability, which include some aspects of evaluating printing accuracies such as extrudability and structural stability (Kadival et al., 2022; Outrequin et al., 2023). For example, rheological properties such as shear-thinning properties, creep relaxations, yield stress, and flow points have been linked to the extrudability and stability of 3D-printed foods (Ma et al., 2021, 2022; Pulatsu et al., 2020; Zhu et al., 2019). A review by Kadival et al. (2022) highlighted that most food printability studies relied on manual measurements of the structure dimensions to evaluate printability. However, automated and quantitative measurement of printing accuracy can compare different printing results and provide feedback for printability improvement. The 3D food extrudability was quantified by Fahmy et al. (2020) using image analysis. The study measured over- and under-extrusions of extruded starch-based filaments in 2 dimensions. Similarly, Zhu et al. (2019) also used video-based measurement to quantify the stability of 3D-printed tomato puree structures. On top of image-based methods, X-ray tomography (XRT) was also used in several studies to measure the internal porosity and infill distributions of 3D-printed foods (Agarwal et al., 2022; Derossi et al., 2021). Although the aforementioned studies provide some quantification of printing accuracy, no automated measurement or direct comparison was made between the original digital designs and the resulting printed foods.

In contrast to 3D printing of food, 3D printing of thermoplastics already utilizes image analysis techniques to in-situ monitor printing accuracy. Petsiuk and Pearce (2020) developed a layer-wise monitoring tool to assess the 3D printing quality and track possible printing errors. A follow-up study illustrated the comparison of the measured image data to digitally rendered structures to detect printing anomalies (Petsiuk and Pearce, 2022). Such a smart monitoring system improves the printing accuracy by identifying the causes of anomalies obtained from the automated analysis. Piovarci et al. (2022) developed a closed-loop control system for direct ink writing printers to avoid over- and under-extrusion using image-based reinforcement learning. As food manufacturing is moving towards digitalization and smart processing, various machine vision techniques have been developed for process monitoring and quality evaluation purposes (L. Zhu et al., 2021). Specifically for 3D food printing, digital image analysis can be promising to quantify printing errors and improve the printing efficiency. Previously, we demonstrated the prediction of food extrudability based on grey-box modeling and the optimization of printing parameters based on computer vision of the extrusion flow rate (Ma et al., 2021, 2023).

Beyond digitally assessing the printing accuracy, human evaluations of the printed food structures are even more important in the context of 3D food printing. Previous sensory studies focused on the sensorial modifications of textures and flavors of 3D-printed foods, while the visual printing accuracy was not often studied extensively in relation to the desirability of consuming 3D-printed foods. An exception is the work of Chirico Scheele et al. (2022) which reported the consumer-rated printing accuracy using a small panel (N = 28). The study showed that participants preferred food structures that closely resembled the digital design, and an increase in the complexity of the printed shape led to higher preference scores. In a follow-up study by the same group (Chirico Scheele et al., 2023), it reported a positive correlation (R^2^ = 0.78) between 3D-printed mashed potato fidelity and desirability of consumption based on an online survey with 50 participants. The study also compared manual shape dimension measurements to the survey fidelity rating and suggested that more variations were found in the survey results than in the manual dimension measurements of the 3D-printed shapes. Therefore, improving 3D printing accuracy can increase desirability of consumption, which is an important first step towards consumer acceptance of 3D-printed foods in the mass market. Furthermore, feedback from manual evaluations can contextualize errors obtained from the dimensional measurements. When developing a digital printing accuracy measurement tool, human evaluations can enhance the applicability of digital methods by contextualizing measurement errors and establishing the relevant tolerance of printing inaccuracy for automated monitoring purposes.

In this study, we aim to develop a layer-wise printing accuracy measuring tool based on automated image analysis and contextualize the digital measurements with human evaluations to establish relevant error tolerance. Cookie dough with different fat sources was 3D-printed and used as example materials for method development purposes. Cookie dough was chosen because it is a common 3D food printing material which also requires post-processing. The layer-wise printing accuracy before and after baking was digitally measured by comparing the 3D-printed cookies to the digital design. An online survey collected visual evaluations of the layer-wise printing accuracy. The survey results and digital assessments were combined to gain insights about printing accuracy and provide guidance to improve future 3D food printing applications.

## 2 Materials and methods

### 2.1 Preparation of printing materials

Wheat flour (Molen de Hoop, the Netherlands), powdered sugar (Van Gilse, the Netherlands), coconut fat (Royal Green, the Netherlands), shortening (Crisco, USA), vegetable oil (Crisco, USA), and butter (Campina, the Netherlands) were purchased from local and online retailers in the Netherlands. Three types of cookie dough composed of different fat ingredients were prepared by using a cookie recipe modified from Pulatsu et al. (2020). Briefly, to prepare 100 g of the cookie dough, i) 20 g of coconut oil, ii) 20 g of shortening, or iii) 10 g of butter and 10 g of vegetable oil was mixed with 17 g of powdered sugar for 5 min at low mixing speed using a kitchen mixer (Kenwood KVL4100, UK), respectively. Then, 49 g of wheat flour was added into the mixture and mixed for 2 min at the same speed. Finally, 14 g of tap water was gradually added into the mixer to form the cookie dough. The cookie dough was mixed for an additional 3 min to obtain a homogenous texture. After mixing, the cookie dough was rested for 10 min at 20 °C prior to the 3D printing experiment. Table 1 provides an overview of the formulations used in this study.

**Table 1:** Formulation overview of the cookie dough used in this study.

Ingredients	Coconut fat cookie	Shortening cookie	Butter and oil cookie
Fat	20 g coconut fat	20 g shortening	10 g butter +10 g vegetable oil
Wheat flour	49 g	49 g	49 g
Tap water	14 g	14 g	14 g
Sugar power	17 g	17 g	17 g

### 2.2 3D printing and post-processing

**Fig. 1:**
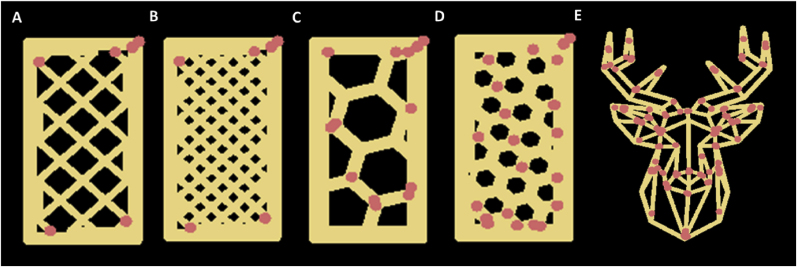
Digital representations of the printing designs. A: rectilinear with 20% infill. B: rectilinear with 40% infill. C: honeycomb with 20% infill. D: honeycomb with 40% infill. E: geometric deer. The red dots indicate a start/stop point of the printing extrusion on the printing path. (For interpretation of the references to color in this figure legend, the reader is referred to the Web version of this article.)

Two digital designs (i.e. block and deer) were sliced using the Slic3r software (https://slic3r.org/) to generate five different printing paths in the form of G-codes. For the block design (50 × 25 × 10 mm), four different printing paths were generated. In specific, 2 infill patterns (rectilinear and honeycomb) and 2 infill levels (20% and 40%) were chosen to make 4 printing paths: rectilinear with 20% infill (RL20), rectilinear with 40% infill (RL40), honeycomb with 20% infill (HC20), and honeycomb with 40% infill (HC40). The deer design was sliced with 0% infill to only print the outline of the geometric design. The digital representations of the printing designs are shown in Fig. 1.

The cookie dough was carefully transferred into a syringe and printed into the designs based on the corresponding G-codes using an extrusion-based 3D food printer (Procusini 5.0, Germany). The cookie dough was printed onto a calibration plate with visual markers for subsequent image analysis purposes. A retraction of 2 mm was used to avoid material oozing at the start/stop points. The start/stop points are the points where the continuous extrusion was started or stopped (see Fig. 1). The printing speed was kept constant at 20 mm/s and a nozzle size of 1.2 mm was used throughout all printing experiments. Each printing geometry was printed in duplicate with all three cookie dough formulations listed in Table 1. In total, 30 cookie samples were printed (3 formulations × 5 designs × 2 repetitions). After printing, the cookies were baked in a convection oven (Heraeus, Germany) at 200 °C for 15 min. Top-view images (from 50 cm above) of freshly printed and baked cookies were acquired using a digital camera (Oneplus Nord, China).

### 2.3 Human evaluations of printing accuracy

**Fig. 2:**
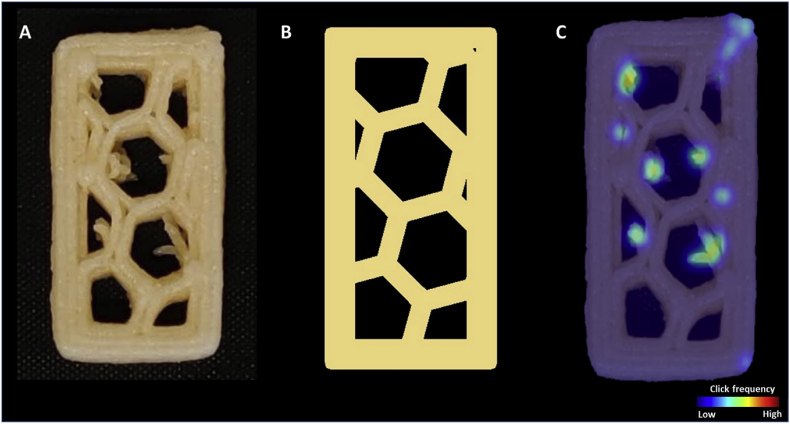
Examples of images used in the online survey and a representative result. A: top view of the printed object (20 × 50 mm). B: Digital design of the printed object. C: Click frequency heatmap obtained from the survey.

An online survey was designed to collect human evaluations of the 3D-printed cookies. To obtain representative results, 5 freshly printed and 5 baked shortening cookies were chosen to be evaluated in the survey. The first part of the questionnaire collected the participants’ demographic information and familiarity with 3D food printing. For the second part of the survey, 10 sets of 2 images (examples shown in Fig. 2A and B) were presented to the participants in random order. Within each image set, the first image was the top view image of the printed object, and the second image was the corresponding digital design of the printed object regenerated using G-codes (see section 2.4.2). A printing line width of 1.5 mm was used to generate all the digital layer images. This line width was set to be slightly larger than the nozzle size (1.2 mm) because expansion of the extruded filament was expected (Ma et al., 2021). The participants were asked to rate the overall accuracy of the printed object in reference to the digital design, using a range from 0 to 100. The left extreme (0) was labeled as “not accurate” while the right extreme (100) was labeled as “very accurate”. Subsequently, the participants were asked to identify the printing inaccuracies by clicking on the image of the printed object. A minimum of 3 clicks and a maximum of 10 clicks were required. In total, 101 respondents completed the survey, and the survey results were visualized using a click frequency heatmap (Fig. 2C). To generate the heatmap, the images of the printed cookie were divided into grids of 9 × 9 pixels, and the number of inaccurate positions was counted within each grid. The frequency values in the grids were then passed through a Gaussian filter to smooth the frequency distribution across neighboring grids. The smoothed frequency values were then visualized with a heatmap to identify the printing inaccuracies perceived by the participants.

### 2.4 Automatic image analysis of printing accuracy

#### 2.4.1 Image acquisition and preprocessing

**Fig. 3:**
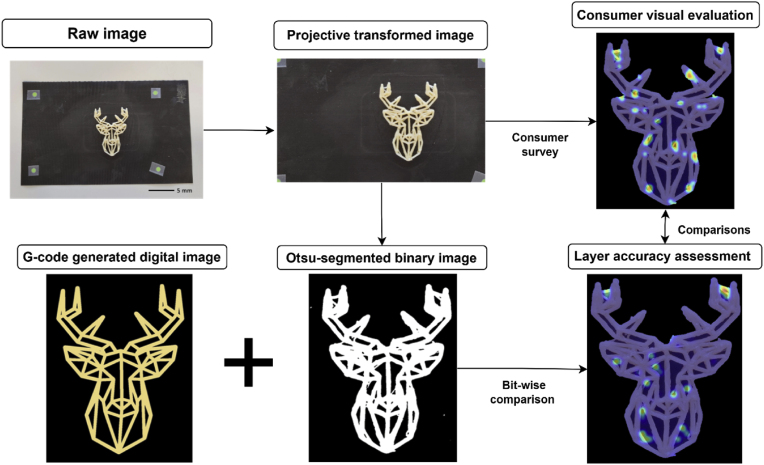
Overview of the automated image analysis pipeline to assess layer-wise printing accuracy and comparison to human evaluations.

Image analysis was used to digitally assess the printing accuracy using the same top-view images as used in the online survey. An automated image analysis pipeline (Fig. 3) was developed to generate digital layer images based on the G-code, quantify the over- and under-extrusion of the printed cookies, and calibrate the digital assessment based on results from human evaluations. Prior to the analysis pipeline, the raw image (4000 × 3000 pixels) was first automatically cropped and perspective transformed to match the reference frame (240 × 150 mm) as marked by the 4 green visual markers. The cropped and transformed image (2400 × 1500 pixels) was scaled using the known distance between the visual markers. The Otsu automatic thresholding method was applied to segment the printed object from the background (Otsu, 1979).

#### 2.4.2 Digital layer image generation

**Fig. 4:**
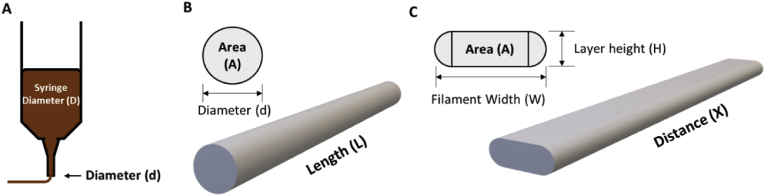
Schematic illustration of the filament width estimation. A: schematic illustration of an extrusion-based food printhead. B: ideally extruded filament. C: filament that was extruded onto a flat surface.

The G-code of the design was parsed into cartesian coordinates of x, y, and z positions of each movement. The corresponding feed rate (F) and extrusion distance (E) were also obtained from the same G-code file. All pairs of motions (X, Y, Z) were connected to iteratively generate the digital layer image. The width of each line was calculated by approximating the cross-sectional area of the extruded filament as a flattened tube. Fig. 4A shows the ideally extruded filament in a cylindrical shape with a cross-sectional circle (⌀ d) and a given length of L. Upon printing onto a surface, the ideally extruded viscous printing material was flattened into a tube, of which the cross-sectional area can be approximated as a rectangle with 2 semi-circular edges (Ma et al., 2021). Based on the conservation of extrusion volume, the filament width (W) from point A (x_1_, y_1,_ E_1_) to point B (x_2_, y_2,_ E_2_) is calculated with:

Eq. 1 $$W=\frac{\pi \Delta ED^{2}}{4XH}+\frac{3}{4}\pi H$$

where $X=\sqrt{{\left(x1-x2\right)}^{2}+{\left(y1-y2\right)}^{2}}$, ΔE = E_2_ – E_1,_ H is the layer height, and D is the syringe diameter.

The pairs of printing motions were then iteratively drawn as connected lines to generate the digital layer image based on the scaling factor of the segmented object image.

#### 2.4.3 Layer-wise printing accuracy assessment

The layer-wise image analysis method provides three ways to quantify the printing accuracy, namely the solid infill, the degree of over-extrusion, and the degree of under-extrusion. The infill (%) was measured for the block designs. To measure the infill, the filled pixels of the segmented object image were counted and divided by the total number of pixels within the block structure. The degree of infill quantifies how closely the printed object resembles the designed degree of infill, which directly impacts the texture of the printed foods.

Over- and under-extrusion were measured from top-view images of the freshly printed cookies, shift factors (in x and y directions) between the digital layer image and the segmented object image were obtained by cross-correlating the two images. The G-code-based layer image was shifted to match the center of mass of the segmented object image. Bitwise logical operations were then performed to examine the degrees of over- and under-extrusion of the printed object relative to the G-code designs. The areas of under-extrusion and over-extrusion are calculated by Eq. (2) and Eq. (3), respectively.

Eq. 2 U=[P|G]⊕P

Eq. 3 O=[P⊕G]⊕U

**Fig. 5:**
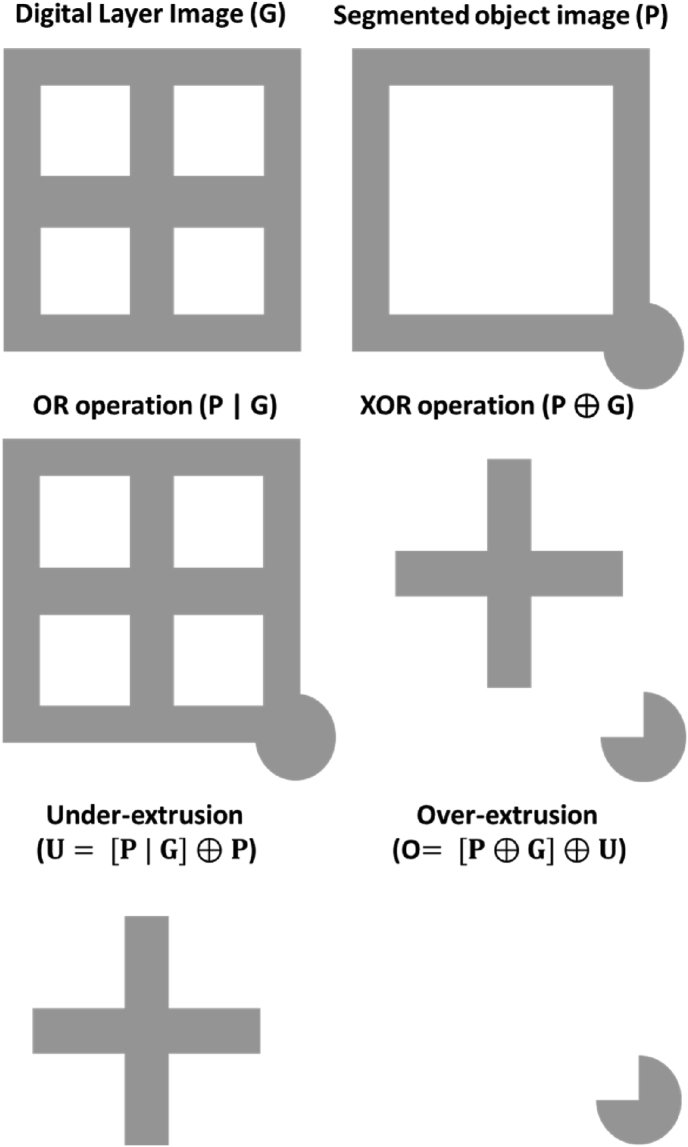
A schematic illustration of the OR and XOR logical operation to extract the over- and under-extrusion areas. The ‘⊕’ represents the XOR logical operation and ‘|’ represents the OR logical operation.

Where *O* and *U* represent the binary images of the over-extruded and under-extruded areas, respectively. *P* is the segmented object image, and *G* is the shifted digital layer image based on the G-code. The ‘⊕’ represents the XOR logical operation and ‘|’ represents the OR logical operation. An illustration of the pixel-wise comparison is shown in Fig. 5.

Preliminary analysis showed that under-extrusion occurs consistently across the complete printed structures, while over-extrusion is more sporadic. To further localize the over-extruded positions on the printed object, the over-extruded proportions were calculated within 9 × 9 grids to evaluate the local printing accuracy. With *n* grids*,* the *O*_*n*_ were calculated using Eq. (3) Over-extrusion indices (i_over_) were calculated based on Eq. (4).

Eq. 4 $$i_{over\;n}=\frac{\sum O_{n}\;\left[o=1\right]}{\sum P_{n}\;\left[p=1\right]+1}$$

Where *n* represents the *n*th grid in the original image and *p* represents the pixels within the P_n_ image grid. The lists of indices (i_over_) of each image were further normalized using a modified percentile normalization technique (Eq. (5)) to account for extreme values calculated from Eq. (4). The normalized i'_over_ was visualized with a heatmap as the initial evaluation of the printing accuracy assessment.

Eq. 5 $$\left\{ \begin{array}{c} if\;i>\;q_{I},99\%i^{\prime }=1\\ if\;i=<\;q_{I},99\%i^{\prime }=\frac{i-\min\left(I\right)}{q_{I},99\%-\min\left(I\right)} \end{array}\right.$$

Where i' is the normalized over- or under-extrusion index, $q_{I},99\%$ is the 99th percentile of I_over._

Since baking may induce additional layer dimension changes, the same layer printing accuracy measurements were also performed on the images of baked cookies. “Incompletion from design” was quantified based on Eq. (2) by comparing the digital layer image and the segmented baked cookie image. Similarly, “expansion from design” was calculated based on Eq. (3) and visualized using Eq. (4) &5 by comparing the digital layer image and the segmented baked cookie image. The changes between over-extrusion and expansion from design and the changes between under-extrusion and incompletion from design were compared to evaluate the impact of baking to layer printing accuracy.

### 2.5 Data analysis and software availability

Pair-wised t-tests, analysis of variance, and Pearson’s correlation tests were conducted using the R programming language. The automated image analysis software was developed using the OpenCV, NumPy, Pandas, and Scikit Image libraries in the Python programming language. The software including a graphical user interface, raw scripts, 3D designs, and the online survey can be accessed through https://git.wur.nl/yizhou.ma/layer-wise-printing-accuracy.

## 3 Results & discussions

In the following sections, the human evaluation of the printing accuracy is introduced to establish the baseline of the printing accuracy of the 3D-printed samples prepared in this study. Then, printing accuracy measurements from automated image analysis are present and contextualized with the results from human evaluations to establish measurement relevance and error tolerance.

### 3.1 Human evaluations of printing accuracy

**Fig. 6:**
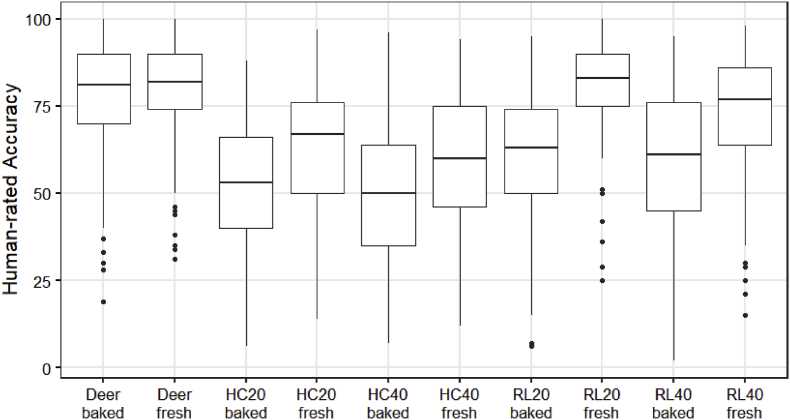
Human-rated printing accuracy of the shortening cookies. HC20 = honeycomb pattern with 20% infill, HC40 = honeycomb pattern with 40% infill, RL20 = rectilinear pattern with 20% infill, and RL40 = rectilinear pattern with 40% infill.

**Fig. A1:**
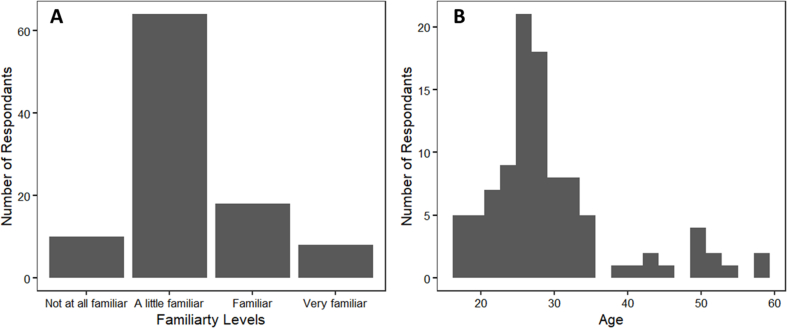
Familiarity levels (A) and age (B) distributions of the survey participants.

In total, 101 valid survey results were obtained from the online questionnaire. The participants consisted of 63 female, 36 male, and 2 non-binary persons with a mean age of 29 years old. Because the survey participants mostly came from food research institutions and companies, 91% of the participants had at least heard about the concept of 3D food printing (i.e. ‘a little familiar’) (Fig. A1). Fig. 6 summarizes the human-rated printing accuracy results. Overall, some variation in the accuracy ratings was observed across all samples. Because the participants of the online survey were untrained, no reference or calibration was done to prime the accuracy towards a certain range. Nevertheless, for each of the block designs, the freshly printed cookies scored significantly higher in printing accuracy compared to the baked ones (P < 0.05). Baking induces a series of physicochemical changes in cookie dough, including water evaporation, fat melting, and starch gelatinization (Pulatsu et al., 2020). As a result of baking, cookies generally spread, collapse in height and their structure deforms (Kim et al., 2019). The dimensional changes negatively impacted the human-rated printing accuracy because they result in further shape deviations from the digital designs. However, for the deer structure, no accuracy difference was observed between the fresh and baked samples, possibly due to the complex structural design, which contains less of the regular geometric elements such as grids and hexagons, in which printing inaccuracies are easily detected. Visual food design complexity has shown to improve food attractions and consumer liking in fine dining and children dining settings (Hamilton et al., 2018; Michel et al., 2014). Therefore, printing more complex structures may alleviate the negative impact of baking by offering more attractive food designs. Meanwhile, according to Chirico Scheele et al. (2022), higher shape complexity also leads to higher consumer preference for 3D-printed foods. Simple geometries (such as the blocks in this study) may be more suitable to evaluate printing stability of food materials in laboratory settings for formulation/process optimization purposes. However, for the perception by consumers, the 3D printing of complex and customized food shapes might be more suited.

**Fig. A2:**
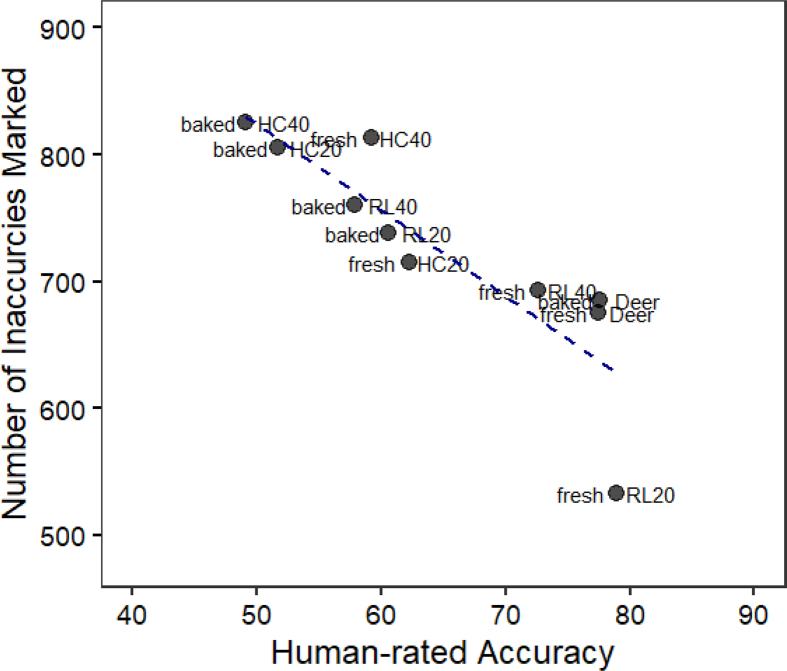
Consumer-rate 3D printing accuracy vs. total number of 3D printing inaccuracies marked per image by survey participants. Correlation = 0.88.

In the same survey, the inaccurate printing positions were also marked. A strong inverse correlation (r = −0.86) was found between the total number of inaccurate positions and the human-rated accuracy, indicating that participants marked more inaccurately printed positions on images with a lower accuracy score (Fig. A2). After confirming the validity of the online survey, the inaccuracies were visualized and overlayed with a digital layer image of the original design. Human evaluations captured printing inaccuracy caused by two typical printing defects, namely material oozing and over-extrusion at the start/stop points.

**Fig. 7:**
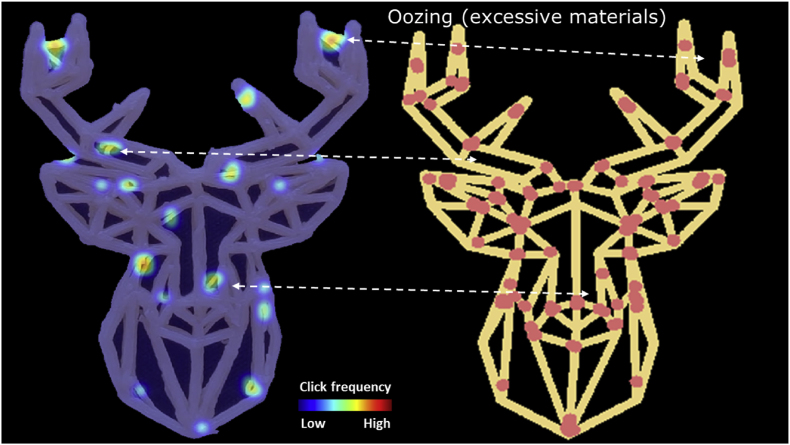
Printing inaccuracies (e.g. oozing) indicated by human evaluations of the freshly printed deer-shaped cookie. The dashed arrows indicate the oozing effects. The red dots represent the start/stop points of a printing layer. (For interpretation of the references to color in this figure legend, the reader is referred to the Web version of this article.)

Fig. 7 shows a representative example of the inaccuracy heatmap generated by the visual evaluations of the questionnaire participants. Some of the inaccuracies can be related to material oozing, which is a common defect in 3D printing of soft materials such as hydrogels and foods (Fahmy et al., 2020; Keong and Hua, 2018). Oozing occurs when the printing flow is not effectively stopped due to material elasticity and/or low viscosity. As observed in Fig. 7, the oozed positions appear to be in between start/stop points on a printing path. During printing, an excess of material flows out of the nozzle when the print head travels to the next start/stop point. In printing parameter optimizations, oozing can be reduced by adjusting the retraction length of the print head (i.e. in the G-codes). For example, Liu et al. (2018) specifically optimized the retraction length to compensate for the oozing of a potato/strawberry juice gel during printing.

**Fig. 8:**
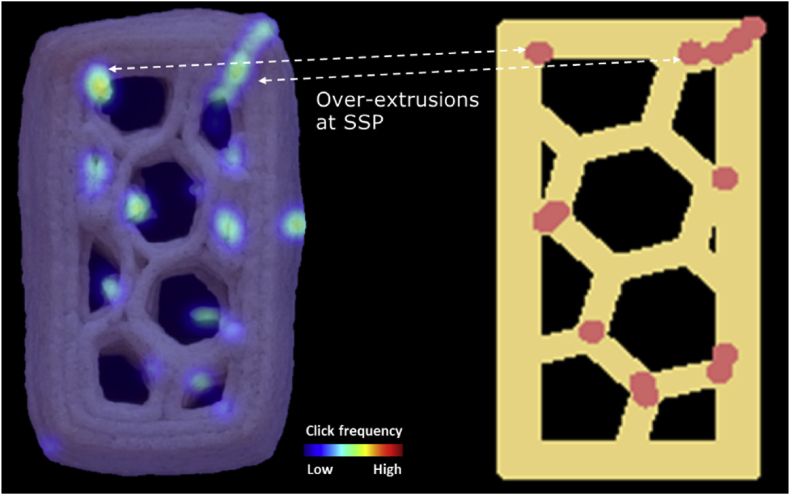
Printing inaccuracies (over-extrusion at start/stop points) indicated by human evaluations of a baked cookie with a 20% honeycomb infill pattern (20 × 50 mm). The dashed arrows indicate the over-extrusion effects. The red dots represent the start/stop points of a printing layer. (For interpretation of the references to color in this figure legend, the reader is referred to the Web version of this article.)

Another representative type of inaccuracy marked manually is over-extrusion at the start/stop points (Fig. 8). The extrusion pressure and flow need to be equilibrated at the beginning or at the end of the extrusion, which leads to variations in material flow (Ma et al., 2023). For the 3D-printed cookies featured in this study, a consistent 2-mm retraction at a speed of 60 mm/s was set in the printing settings. In reality, the speed of the retraction was not fast enough to rapidly stop the extrusion flow, which can lead to over-extrusion of the material at the stop point of a continuous extrusion path. At the start of an extrusion path, the retracted 2 mm needs to be compensated by quickly bringing the piston down, which can lead to an over-extrusion at the beginning of a printing segment. The over-extrusion at the start/stop points can be explained by the shear-thinning behavior of the cookie dough. A rapid push of the piston increases the pressure in the syringe, which then reduces the printing material’s viscosity, leading to the observed over-extrusion shown in Fig. 7. For 3D printing of soft materials, a continuous extrusion of the printing materials across the printing path is therefore preferred over having multiple start/stop segments (Fernandez et al., 2019). Our results from the survey support this finding. Thus, optimizing the printing path by using continuous extrusion may improve the printing accuracy as perceived by human evaluators.

Overall, the printing inaccuracies marked by survey participants are related to over-extrusion defects rather than those of under-extrusion. This is likely due to that we surveyed layer printing accuracy of the final printed shape. In 3D food printing applications, under-extrusion of printing materials often occurs at the beginning of an extrusion and is more observable when printing single layers (Fahmy et al., 2020; Ma et al., 2023). During 3D printing of multiple layers, the under-extruded portions can be covered by subsequent layers, which makes under-extrusion less recognizable from the top-view. In contrast, over-extrusion is more observable and directly comparable with the digital design using the automated image analysis measurement. Therefore, a quantitative approach is needed to measure the over-extrusion to monitor and control the 3D food printing process and achieve better printing accuracy. The insights from the online survey serve as the basis for the subsequent evaluation of the printing accuracy using the automated digital assessment tool.

### 3.2 Layer-wise digital image analysis of printing accuracy

Layer-wise digital image analysis is used to describe how well the freshly-printed or post-processed food structure resembles the original digital design. In this study, we measured the degree of layer infill (%) before and after baking as a general indicator for the textural modifications. Then, the over- and under-extrusion of the printed cookies were quantified to connect the observed printing inaccuracy to corrective control actions.

#### 3.2.1 Layer infill measurement

**Fig. 9:**
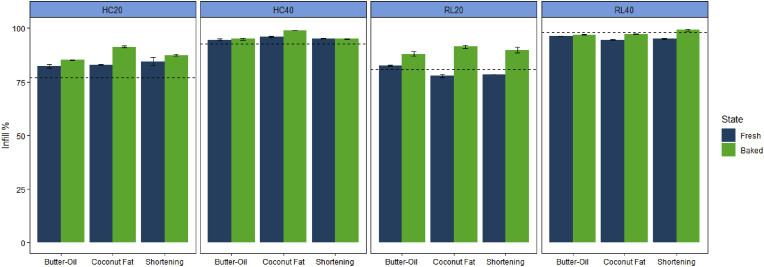
Layer infill % measured by the digital image analysis method developed in this study. HC20 = honeycomb pattern with 20% infill, HC40 = honeycomb pattern with 40% infill, RL20 = rectilinear pattern with 20% infill, and RL40 = rectilinear pattern with 40% infill. The error bar indicates the standard deviation of the averaged infill % (n = 2). The dashed line indicates the degree of infill calculated from the digital layer image obtained from the G-code in section 2.4.2.

The degree of infill of the block designs was first measured based on the layer-wise image analysis method. It determines the porosity of the printed structure, which is a common design parameter to control the textural properties of 3D-printed foods (Fahmy et al., 2022; S. Zhu et al., 2021). Fig. 9 highlights the measured degree of infill of cookies before and after baking by analyzing the corresponding top-view images. Even though the 2D infill setting in the Slic3r software was either 20 or 40%, the measured infill of the digital layer image was 77 or 88%, respectively. The deviation between the software infill settings and the measured infill % shows that the Slic3r software setting is not an accurate estimation of the infill of the true layer. Severini et al. (2016) reported a similar difference between the software infill settings and the solid fractions of the printed object. The authors suggested that the software infill calculation does not include the external shell, which constitutes a large part of the solids in the printed structure. For accurate estimations of the infill % and structural porosity, one should use the digital layer image generated from this study as the reference rather than simply relying on the slicing software settings.

Across the different structures, except for the RL40, most of the printed structures exceeded the degree of infill of the digital design. This is likely due to over-extrusion. For RL40, the designed infill obtained by analyzing the G-code generated image is over 90%, which makes the effect of over-extrusion less apparent. Overall, baking leads to a significant increase in the degree of infill for all structures (P < 0.05). The baking-induced cookie dough spread agreed with the human evaluation results discussed in section 3.1. For textural modification purposes, one should therefore determine the degree of infill of the baked cookies (e.g. or porosity) and how that influences the cookie texture, rather than only relying on the degree of infill of the digital structure or the freshly printed objects. For example, in some previous studies, researchers used X-ray computed tomography to directly measure the true porosity of the 3D objects (Derossi et al., 2021; Severini et al., 2018), which is an effective method to quantify textural modification and structural porosities in laboratory settings. For practical situations, the degree of infill based on our layer-wise image analysis provides 2D infill measurement that can offer rapid and cost-effective estimations suitable for routine 3D food printing applications.

#### 3.2.2 Over-extrusion

**Fig. 10:**
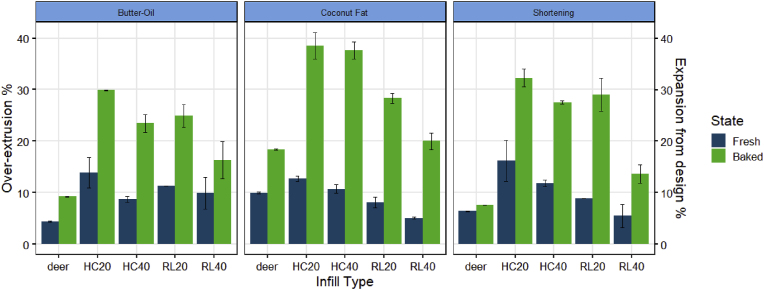
Over-extrusion % (fresh) and expansion from design % (baked) 3D-printed cookies measured by the layer-wise image analysis method developed in this study. HC20 = honeycomb pattern with 20% infill, HC40 = honeycomb pattern with 40% infill, RL20 = rectilinear pattern with 20% infill, and RL40 = rectilinear pattern with 40% infill. The error bar indicates the standard deviation of the averaged over-extrusion % or expansion from design % (n = 2).

For all cookies, the baked version had significantly (P < 0.05) more over-extrusion compared to the freshly printed cookie dough (Fig. 10). The spreading of baked cookies aligned with previous observations from the survey and the measured degrees of infill (section 3.1, and section 3.2.1). The increased over-extrusion after baking leads to reduced accuracy of the printed structures. It is worth noting that all samples exhibit some over-extrusion when compared to the digital designs. The source of this may be the die-swelling during extrusion-based 3D food printing (Le-Bail et al., 2020). Cookie dough is first compressed and extruded through a small nozzle, after which the elastic cookie dough can relax which leads to transversal expansion of the filament. Such a die-swell effect results in wider filaments, which contributes to the observed over-extrusion shown in Fig. 10. No significant difference (P > 0.05) was found among the fat ingredients. While the fats had different melting points, all cookie doughs contained solid fat crystals, and exhibited a similar die-swell during extrusion and spreading after baking.

**Fig. 11:**
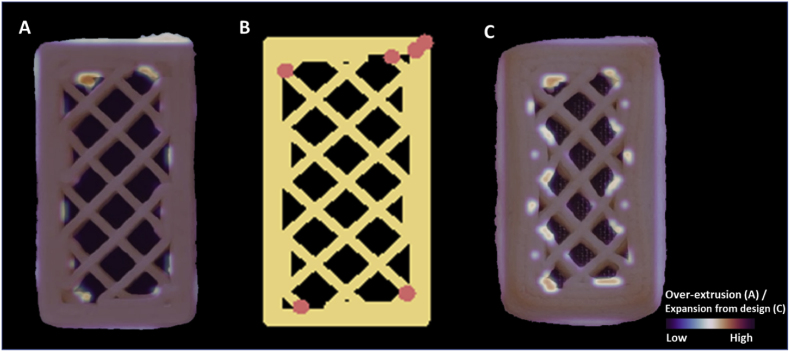
A: over-extrusion visualization of the freshly printed coconut oil cookie. B: digital layer image of the rectilinear pattern and 20% infill (20 × 50 mm). C: expansion from design visualization of the same cookie after baking. The red dots represent the start/stop points of a printing layer. (For interpretation of the references to color in this figure legend, the reader is referred to the Web version of this article.)

Over-extrusion at the level of the whole image provides an overview of the printing accuracy. Using the automated image analysis tool, we can also localize the printing inaccuracies within a given structure similar to the human evaluations discussed in section 3.1. These localized printing inaccuracies may identify the cause of the printing defects and help to eventually improve the printing paths to minimize the inaccuracies. Fig. 11A highlights the over-extruded positions of a freshly printed coconut oil cookie (RL20) on a heatmap. The over-extrusions are sparse across the rectilinear block structure, rather than distributed evenly. Compared to the digital layer image (Fig. 11B), the measured over-extruded positions overlap with the start/stop points. This observation agrees with the human evaluations, in which participants also marked the over-extrusions at the start/stop points. The automated layer-wise image analysis therefore can capture over-extrusion similar to human evaluations. In addition, some oozing is identified in Fig. 11A, which aligns with the survey results discussed in section 3.1. Both human evaluations and automated image analysis suggest that over-extrusion is likely to occur when the extrusion flow is disrupted (e.g. at start/stop points).

When evaluating the same cookie before and after baking, many more over-extruded positions are identified in Fig. 11C. These all appear between the external shell and the infill lines, indicating some structural deformation during baking (i.e. the external shell collapsed towards the pore-filled center). In general, a decrease in height and an increase in width and length by baking is observed (Kim et al., 2019). As shown in Fig. 10, after baking, the layer-wise over-extrusion increases for the RL20 coconut cookie. The reduced printing accuracy of a 3D printed food after processing is consequently not only determined by the printing process, but also by the post-processing treatment (Petsiuk and Pearce, 2020). Corrective control actions such as reducing the printing speed and adjusting the baking parameters may alleviate the observed over-extrusion during printing or post-processing.

Overall, the automated image analysis provides comparable over-extrusion results as human evaluations of the freshly-printed samples. The human evaluations contextualize the digital measurements, which support that human assessment and digital image analysis recognize the same level of printing inaccuracy in terms of over-extrusion. Digitally quantifying over-extrusion can therefore be applied for in-line monitoring and post-printing screening purposes. When determining the printing accuracy of the final product, post-processing parameters should also be considered as structural changes such as collapse and deformation are likely to happen during post-processing. By routinely monitoring the printing accuracy of freshly printed and post-processed structures, future studies may be able to connect post-processing parameters to printing accuracy, which can use the digital image data obtained from this study for further process optimization purposes.

#### 3.2.3 Under-extrusion

**Fig. 12:**
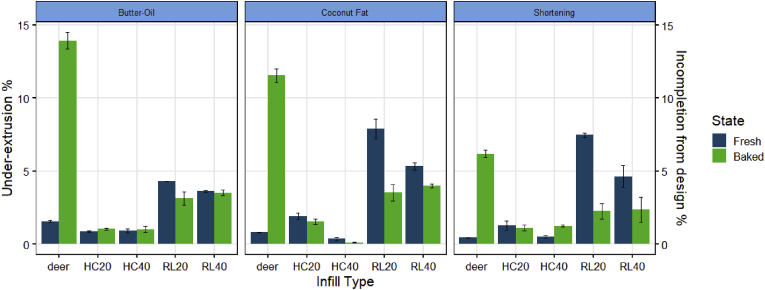
Under-extrusion % (fresh) and incompletion from design % (baked) 3D-printed cookies measured by the layer-wise image analysis method developed in this study. HC20 = honeycomb pattern with 20% infill, HC40 = honeycomb pattern with 40% infill, RL20 = rectilinear pattern with 20% infill, and RL40 = rectilinear pattern with 40% infill. The error bar indicates the standard deviation of the averaged under-extrusion % or incompletion from design % (n = 2).

While over-extrusion is an apparent indicator of inaccurate printing, under-extrusion provides another aspect of layer accuracy characterization. Fig. 12 highlights the measured under-extrusion for all the 3D-printed cookies. Overall, the extent of under-extrusion (up to 15%) is less than the extent of over-extrusion (above 30%). In contrast to the deer design, the block designs consistently had less than 8% under-extrusion. This lower degree of under-extrusion matches with our earlier speculation that 3D printing operators may have some selection bias to avoid printing with little or no extrusion flow.

**Fig. 13:**
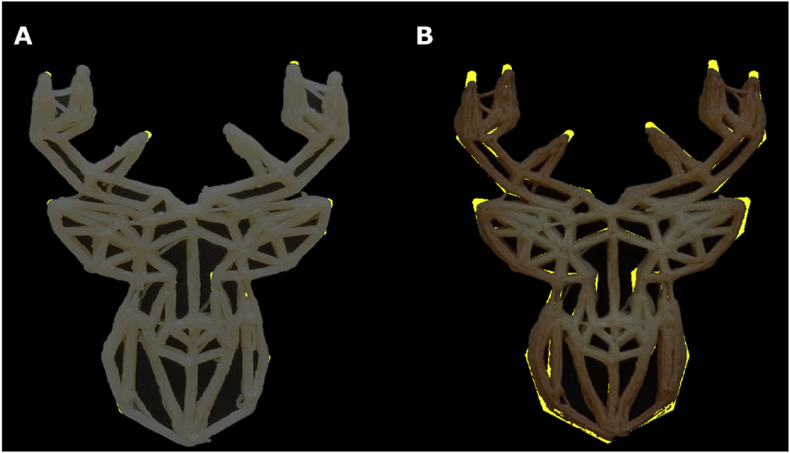
Under-extrusion visualizations of freshly printed (A) and baked (B) deer structures printed with the shortening cookie dough. The yellow color represents the missing position of the print in comparison to the digital design. (For interpretation of the references to color in this figure legend, the reader is referred to the Web version of this article.)

A general decreasing trend is observed when comparing the under-extrusion of the freshly printed and baked blocks. This decrease in under-extrusion correlates with the increase in over-extrusion as discussed in previous sections, which can be explained by the baking expansion and spread of the cookie dough. However, it is interesting to observe that for the deer design, more under-extrusion is found for both the freshly printed and the baked structures. Because the deer design is a single-line geometrical drawing, the increase of under-extrusion in the freshly printed structure may indicate a misposition between the printed structure and the digital layer image. After extrusion, the single extruded lines are subject to relaxation, which may appear as shrinking or rounding off of the printed shape, causing the misposition as highlighted in Fig. 13A. Due to the larger surface area of the deer structure, the structure may experience more moisture loss during the same baking time, which then causes further shrinking and misposition of the baked deer structure (Fig. 13B). In addition, as shown in Fig. 10, Fig. 12, over-extrusion and under-extrusion can co-exist at the whole image level, emphasizing the necessity to localize the printing inaccuracy and analyze the specific regions of error.

**Fig. 14:**
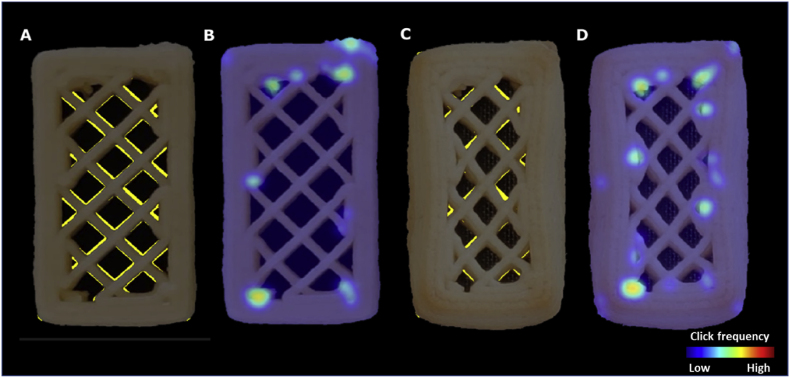
A: visualization of under-extrusion of the freshly printed coconut oil cookie with the rectilinear pattern and 20% infill (20 × 50 mm). B: the same cookie evaluated manually. C: baked coconut oil cookie with the rectilinear pattern and 20% infill (20 × 50 mm). D: the same cookie evaluated manually. The yellow color in A and C represents the missing position of the print in comparison to the digital design. (For interpretation of the references to color in this figure legend, the reader is referred to the Web version of this article.)

Fig. 14 shows some examples of block designs to compare the locations of under-extrusion to the inaccuracies identified by the survey participants. The yellow pixels highlight the missing positions compared to the digital layer image (Fig. 14A). For a freshly printed cookie, consistent under-extrusion is found as the highlighted yellow regions are evenly distributed around the infill lines. The survey participants did not identify such a consistent under-extrusion as printing inaccuracy (Fig. 14B). The human-rated printing inaccuracies are driven by over-extrusion. After baking, the cookie dough spreads and deforms, which reduces some of the under-extrusion as measured via the layer-wise method (Fig. 14C). However, the under-extrusion positions still do not overlap with the human-rated inaccurate positions. Human evaluations do not identify consistent printing accuracy errors such as the under-extrusion observed in Fig. 14A, but rather mostly see irregularities. This illustrates the valuable insights collected from human evaluations, which can help calibrate the digital method to best identify relevant printing defects. Without the human evaluation reference, the layer-wise digital method may measure excessive under-extrusion of a given structure and reject it as an inaccurate print, while consumers would not mind. Contextualizing the various errors in printing accuracy by calibrating with human evaluations is therefore vital to the applicability of the digital method developed in this study.

### 3.3 General discussions

Layer-wise image analysis is a simple and automated monitoring technique to measure 3D printing accuracy. Usually, the target of 3D printing is to replicate digital designs as closely as possible, so any defects (e.g. over- and under-extrusions) should be avoided as much as possible (Piovarci et al., 2022). Printing anomalies (e.g. blocked nozzle, incomplete print) are well-defined and monitored in 3D plastic and metal printing applications (Petsiuk and Pearce, 2022). However, for 3D food printing, there is no standardized definition of printing quality solely based on numerical measurements. Because the ultimate purpose of 3D food printing is to provide customized foods, consumer opinions should be included when performing printing quality assessment. Therefore, a digital assessment tool should be validated with feedback from humans. Even though under-extrusion was frequently detected by the automated image analysis method, the survey participants did not recognize under-extrusion as inaccurate printing in the current study, as long as it occurs consistently across the structure. This observation may be different when the consumers taste printed foods with under-extrusions, as they may have less mechanical strength and carry a different textural property. For the end application of 3D food printing, consumer inputs should define what type of printing accuracy is important, which can then be used to calibrate the digital quality assessment tool(s) developed in this study.

**Fig. 15:**
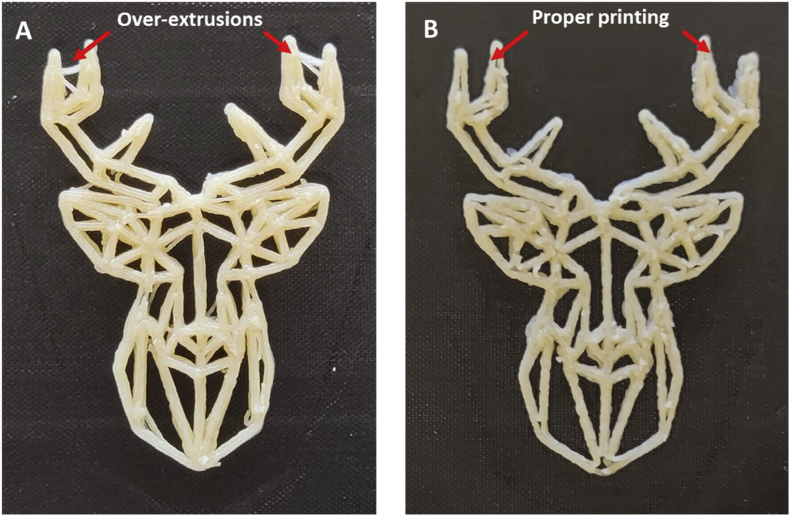
Corrective action showcase. A: Original deer structure printed with the shortening cookie dough. B: Improved deer structure to prevent over-extrusion by increasing the level of retractions. The red arrows indicate the accuracy improvement before and after the corrected retraction. (For interpretation of the references to color in this figure legend, the reader is referred to the Web version of this article.)

Automatically detecting the printing inaccuracies provides indications of 3D-printed food quality, but it will be more beneficial to link the detected inaccuracies to corrective actions during printing. The most effective action, as suggested by Petsiuk and Pearce (2020), is to stop faulty printing jobs when irreversible defects are identified. This automatic shut-down function can certainly save time and printing waste in manufacturing, but more specific actions may increase the adaptability of 3D printing. For example, in Fig. 15, when oozing is detected, additional retractions can be built into the subsequently printed structure (via modification of the G-code), which provides a dynamic amount of retractions based on the image analysis feedback. This adaptive approach is especially helpful when producing multiple structures in a continuous printing series, which is often the case for personalized food productions. Furthermore, quantifying the layer-wise printing accuracy using image analysis can provide insight about the effects of material properties such as shear recovery and creep relaxation. For example, an increase of the printing line width or consistent geometric shrinking can be linked to certain rheological properties of the specific printing materials. Formulation adjustments can then be implemented to minimize the apparent printing errors by including additional functional ingredients. Finally, with such a digital method, one can further optimize post-processing parameters by comparing the freshly printed and post-processed printing accuracy. The automated image analysis method provides rapid feedback to collectively optimize printing material formulation, printing parameters, and post-processing parameters.

Although layer-wise image analysis can provide actionable assessment for 3D food printing, the current monitoring approach comes with some limitations. For all structures featured in this study, no bottom layer was printed to provide a color contrast to segment the printed structure from the background. Therefore, the layer-by-layer assessment is not possible using a single 2D camera. In addition, corrective actions such as increasing the amount of retraction are still manually implemented. Further automation of the 3D food printing process is limited by the current system which does not allow for in-line adjustment or closed-loop control. To address these limitations, future implementations of the layer-wise monitoring method may use high-resolution 3D cameras to capture both the color and depth information of a given layer. Sub-millimeter resolution of depth could enable segmentation of a single layer to allow layer-by-layer printing accuracy assessment. Ultimately, sophisticated printing controllers can be adapted from metal and plastic printers to 3D food printing, which can empower in-line control during 3D food printing.

## 4 Conclusions

In this study, automated layer-wise image analysis was employed to quantify 3D food printing accuracy in terms of over- and under-extrusion. The measured printing inaccuracy was compared to human evaluations to contextualize the digital measurements. Human evaluators identified inaccurate printing aspects as oozing and over-extrusion localized at the start/stop points on a printed structure. The human-rated and digital evaluations both captured over-extrusion of 3D-printed cookies. Baking in general negatively impacts the printing accuracy of 3D-printed cookies with simple geometries. While the digital method captured under-extrusion as a printing defect, human evaluations did not identify consistent under-extrusion as inaccurate printing in the survey. The insight from the human evaluations can therefore guide the digital assessment based on layer-wise image analysis. Based on the digital assessment, corrective actions such as increasing retractions to prevent oozing can be realized to improve the printing accuracy. A digital method guided by human insights can provide simple and automated evaluations of 3D food printing accuracy and provide meaningful guides to improve printing accuracy and efficiency.

## CRediT authorship contribution statement

**Yizhou Ma:** Conceptualization, Methodology, Investigation, Formal analysis, Writing – review & editing. **Jelle Potappel:** Conceptualization, Supervision, Writing – review & editing. **Maarten A.I. Schutyser:** Conceptualization, Supervision, Writing – review & editing. **Remko M. Boom:** Supervision, Writing – review & editing. **Lu Zhang:** Conceptualization, Supervision, Writing – review & editing.

## Declaration of competing interest

The authors declare to have no conflicting interests.

## Data Availability

Data will be made available on request.
